# GABA_A_ receptor drugs and neuronal plasticity in reward and aversion: focus on the ventral tegmental area

**DOI:** 10.3389/fphar.2014.00256

**Published:** 2014-11-25

**Authors:** Elena Vashchinkina, Anne Panhelainen, Teemu Aitta-aho, Esa R. Korpi

**Affiliations:** ^1^Department of Pharmacology, Institute of Biomedicine, University of HelsinkiHelsinki, Finland; ^2^Institute of Biotechnology, University of HelsinkiHelsinki, Finland; ^3^Department of Pharmacology, University of CambridgeCambridge, UK; ^4^Department of Pharmacology, Yong Loo Lin School of Medicine, National University Health System, Neurobiology and Ageing Programme, Life Sciences Institute, National University of Singapore, and SINAPSE, Singapore Institute for NeurotechnologySingapore, Singapore

**Keywords:** GABA_A_ receptor, benzodiazepines, THIP, neurosteroids, dopamine neurons, neuroadaptation, dendritic spines

## Abstract

GABA_A_ receptors are the main fast inhibitory neurotransmitter receptors in the mammalian brain, and targets for many clinically important drugs widely used in the treatment of anxiety disorders, insomnia and in anesthesia. Nonetheless, there are significant risks associated with the long-term use of these drugs particularly related to development of tolerance and addiction. Addictive mechanisms of GABA_A_ receptor drugs are poorly known, but recent findings suggest that those drugs may induce aberrant neuroadaptations in the brain reward circuitry. Recently, benzodiazepines, acting on synaptic GABA_A_ receptors, and modulators of extrasynaptic GABA_A_ receptors (THIP and neurosteroids) have been found to induce plasticity in the ventral tegmental area (VTA) dopamine neurons and their main target projections. Furthermore, depending whether synaptic or extrasynaptic GABA_A_ receptor populations are activated, the behavioral outcome of repeated administration seems to correlate with rewarding or aversive behavioral responses, respectively. The VTA dopamine neurons project to forebrain centers such as the nucleus accumbens and medial prefrontal cortex, and receive afferent projections from these brain regions and especially from the extended amygdala and lateral habenula, forming the major part of the reward and aversion circuitry. Both synaptic and extrasynaptic GABA_A_ drugs inhibit the VTA GABAergic interneurons, thus activating the VTA DA neurons by disinhibition and this way inducing glutamatergic synaptic plasticity. However, the GABA_A_ drugs failed to alter synaptic spine numbers as studied from Golgi-Cox-stained VTA dendrites. Since the GABAergic drugs are known to depress the brain metabolism and gene expression, their likely way of inducing neuroplasticity in mature neurons is by disinhibiting the principal neurons, which remains to be rigorously tested for a number of clinically important anxiolytics, sedatives and anesthetics in different parts of the circuitry.

## INTRODUCTION

GABA_A_ receptor agonists generally depress brain activity. Benzodiazepines (BZs) and other GABAmimetic drugs depress gene expression in the brain, including the neuroplasticity-related genes such as brain-derived neurotrophic factor (BDNF), nerve growth factor (NGF), and Fos-genes (Zafra et al., 1991; Huopaniemi et al., 2004). BZs and volatile anesthetic isoflurane also have been reported to reduce long-term potentiation (LTP) in several brain regions (Higashima et al., 1998; Kulisch et al., 2011; Piao et al., 2013). Moreover, BZs and general anesthetics are able to impair neurogenesis in both young and adult animals (Wu and Castren, 2009; Erasso et al., 2013; Thal et al., 2014). All these facts suggest that GABA_A_ receptor agonists have little potential for neuroplasticity outside the critical periods of brain development, when the role of GABAergic interneurons is obligatory (Hensch and Stryker, 2004). On the other hand, by suppressing the local inhibitory regulation, GABA_A_ receptors on GABAergic interneurons can indirectly cause activation of principal neuron populations, a network effect called disinhibition. For example, GABAergic neurons in the lateral part of the central nucleus of amygdala are excited by low acute doses of BZs through disinhibition and this is associated with anxiolytic effects (Beck and Fibiger, 1995; Salminen et al., 1996; Panhelainen and Korpi, 2012). Furthermore, GABA_A_ receptor agonists can activate the dopaminergic (DAergic) neurons in the ventral tegmental area (VTA) by disinhibition (Heikkinen et al., 2009; Tan et al., 2010; Vashchinkina et al., 2012).

In this review we will discuss recent advances in understanding how GABA_A_ receptor modulators affect both GABAergic and glutamatergic synapses and also how their acute or repeated treatments can modulate plasticity of the reward system. Given the widespread application of GABA_A_ receptor drugs, understanding more fully how they modulate brain reward system may help to develop new strategies for designing novel compounds to overcome their therapeutic limitations.

## GABA_A_ RECEPTORS AND THEIR MODULATORS

Benzodiazepines, inhalational and intravenous anesthetics, barbiturates, neurosteroids, and other GABAmimetic drugs – all share the interaction with the GABA_A_ receptor and facilitation of receptor function to produce strong pharmacological and behavioral actions (Sieghart, 1995; Korpi et al., 2002). They act on distinct sites on the GABA_A_ receptor and increase membrane anion (Cl^-^ and bicarbonate) conductance, thereby in most cases inducing hyperpolarization, which has an inhibitory effect on the firing of the postsynaptic neurons.

The GABA_A_ receptors belong to the “cys-loop” superfamily of ligand-gated ion channels (Alexander et al., 2013). They exist as heteropentameric structures, commonly composed of two α subunits, two β subunits, and one γ or δ subunit (McKernan and Whiting, 1996). The BZ-sensitive GABA_A_ receptors contain either α1, α2, α3, and/or α5 subunits and as a result of containing the γ2 subunit, they are preferentially located at synaptic sites (with the exception of α5), whereas the α4 or α6 subunit-containing GABA_A_ receptors are highly sensitive to neurosteroids, GABAmimetic drugs such as muscimol and THIP (gaboxadol; 4,5,6,7-tetrahydroisoxazolol[4,5-c]pyridine-3-ol), general anesthetics such as isoflurane and etomidate. Since they often possess the δ subunit instead of γ2, they are located at peri- or extrasynaptic sites (Olsen and Sieghart, 2009).

GABA_A_ receptor modulators are effective in the wide range of indications (anxiety disorders, panic, insomnia, muscle spams, seizure control in epilepsy and alcohol withdrawal, sedation of aggressive patients, calming down anxious patients before operations, and induction of anesthesia) by acting on distinct GABA_A_ receptor subtypes which demonstrate a unique heterogeneity in terms of function, kinetics, pharmacological profile, and distribution in brain (Uusi-Oukari and Korpi, 2010). Combination of pharmacological and genetic approaches has revealed that 1) α1 subunit-containing GABA_A_ receptors mainly mediate the sedative and addictive effects of BZs, 2) α2 or α3 subunit-containing receptors mediate the anxiolytic and muscle-relaxant effects, and 3) α5 subunit-containing receptors mediate the memory-impairing effects of BZs (Crestani et al., 1999; Rudolph and Mohler, 2004). Furthermore, there is growing evidence that hippocampal extrasynaptic α5 subunit-containing receptors contribute to amnestic effects by general anesthetics (Grasshoff et al., 2005), while the stimulation of δ subunit-containing receptors mediate anxiolytic, anticonvulsive, anesthetic, and aversive effects of neurosteroids in mice (Mihalek et al., 1999; Vashchinkina et al., 2014).

Despite the usefulness of GABA_A_ receptor modulators, their use may lead to side-effects that limit their efficacy. General anesthetics may induce postoperative cognitive dysfunction for weeks or months in elderly patients (Canet et al., 2003; Newman et al., 2007), and their use in prolonged operations at neonatal period, although necessary, have raised serious thoughts about possible neuronal damage and long-term cognitive effects as seen in preclinical models (Jevtovic-Todorovic et al., 2013). Beside the short-term undesirable effects of BZs such as dizziness and motor impairment, their long-term effects include disruption of sleep architecture, confusion and memory impairment, tolerance and dependence (Licata and Rowlett, 2008). Furthermore, chronic increased exposure to neurosteroids appears to accelerate development of Alzheimer’s disease symptoms in various mouse models (Bengtsson et al., 2012, 2013). Thus, the use of these drugs causes persistent neuroadaptation in the brain that also may contribute to adverse effects.

## GABA_A_ DRUGS IN THE ANIMAL MODELS OF ADDICTION

Addiction is increasingly seen as a disease of aberrant neuroadaptation in the brain reward system (Volkow and Baler, 2014). The VTA dopamine neurons are considered as an essential hub at least for the early phases of addiction. VTA has reciprocal connections with many forebrain centers such as the nucleus accumbens (NAc, ventral striatum), dorsal striatum, the medial prefrontal cortex (mPFC), the extended amygdala (particularly including the basolateral (BLA) and central nuclei of amygdala and the bed nuclei of stria terminalis) and lateral habenula, which circuitries form the major part of reward and aversion pathways.

The risk of developing addiction is one of the main challenges restricting the clinical use of BZs and other GABA_A_ modulators (O’Brien, 2005). Addiction-related behaviors can also be studied in experimental animal models. Drugs of abuse activate the reward system and produce reinforcing effects in drug self-administration, in potentiation of the intracranial electrical self-stimulation reward, and in drug-induced learning and conditioning. Various non-selective BZ agonists, such as diazepam and midazolam, are self-administered (Tan et al., 2010). They are rewarding in conditioned place preference paradigm (Spyraki et al., 1985) and able to increase the rate of responding for and to decrease the threshold of the intracranial self-stimulation reward (Straub et al., 2010). The α1 subunit-preferring agonist zolpidem is self-administered in baboons (Ator, 2002), but produces no place preference in rats (Meririnne et al., 1999), showing thus somewhat unclear effects. This might be due to zolpidem’s strong dose-dependent sedative effect. The α2 subunit-containing GABA_A_ receptors in the NAc were necessary for the midazolam preference in a test giving the mice a choice between sucrose solution and sucrose/midazolam solution (Engin et al., 2014). The endogenous neurosteroid GABA_A_ agonist allopregnanolone displays variable effects in animal models of addiction. It can maintain oral self-administration, although not in operant setting (Sinnott et al., 2002). It decreases the threshold of the intracranial self-stimulation reward (Fish et al., 2014), and when injected intraperitoneally (ip), it has been shown to induce either rewarding (Finn et al., 1997) or aversive conditioning effects (Beauchamp et al., 2000). Propofol is rewarding in conditioned place preference (Pain et al., 1996), while isoflurane and GABA_A_ β2/3 subunit-selective etomidate have not been tested so far.

In attempts to further localize the reward-related effects of GABA_A_ modulators in brain circuitry, intracerebral drug infusions have been used. GABA_A_ agonist muscimol and antagonist bicuculline show interesting effects as they both are reinforcing and rewarding in drug-naïve animals when infused into the VTA (Ikemoto et al., 1997, 1998; Laviolette and van der Kooy, 2001). These results have been explained as effects arising from two reward systems, one being DAergic and the other non-DAergic. Muscimol is believed to inhibit VTA GABA neurons and thus disinhibit VTA DA neurons leading to DA antagonist-sensitive reward, while bicuculline also acts on VTA GABA neurons but activates them to target a poorly defined non-DAergic reward pathway insensitive to DA receptor antagonists (Laviolette and van der Kooy, 2001). It remains to be studied, e.g., whether bicuculline acts on VTA GABA neurons projecting to NAc cholinergic interneurons, thereby potentiating appetitive associative learning (Brown et al., 2012). These intracerebral infusion studies should be replicated by regionally and neuronally more specific methods such as chemogenetics or optogenetics to avoid possible confounding infusion-site diffusion to other regions and to various neuronal populations. Furthermore, the brain circuitry for reward and aversion goes well beyond the VTA, stressing the importance of systemic drug experiments for translational relevance.

## PLASTICITY OF GLUTAMATERGIC AND GABAergic SYNAPSES

Growing evidence indicates that neuroadaptation (plasticity) in the brain appears in both glutamatergic and GABAergic synapses (for review, see Castillo et al., 2011; Luscher and Malenka, 2011; Kullmann et al., 2012). Synaptic connections are highly plastic and constantly modified by environmental factors and learning tasks. It should be noted that generation of new glutamatergic and GABAergic synapses proceeds under distinct mechanisms with different factors regulating the processes (described below).

The major sites of contact for glutamatergic presynaptic terminals are dendritic spines (Gray, 1959; Dailey and Smith, 1996; Hering and Sheng, 2001), and dynamic changes in the morphology of dendritic spines have been associated with changes in synaptic strength (Segal, 2005). Spine morphology is subject to rapid alteration by patterns of neuronal activity and by activation of postsynaptic glutamate receptors (Lang et al., 2004; Matsuzaki et al., 2004). Thus, synaptic efficacy can be regulated by multiple mechanisms.

Conversely, the predominant sites of contact for GABAergic presynaptic terminals are not located on spines, but directly on dendritic shafts (Freund and Buzsaki, 1996; Somogyi et al., 1998). However, GABAergic system also interacts with synapses on spines. Recent work has indicated that the activity of GABA_A_ receptors affects spine maturation (Heinen et al., 2003; Jacob et al., 2009). In rat hippocampal cultures, reduced GABA_A_ receptor endocytosis (and increased activity) is associated with reduced spine maturation and reduced levels of postsynaptic density protein-95 (PSD-95; Jacob et al., 2009), and in the visual cortex of adult GABA_A_ receptor α1 subunit knockout mice (resulting in compensatory increased inhibition), the spine density is reduced with a corresponding reduction in PSD-95 expression (Heinen et al., 2003), results of both studies being in agreement with GABA_A_ receptor activation reducing LTP (Higashima et al., 1998). Another study (Shen et al., 2010) showed that development-dependent expression of α4 subunit-containing GABA_A_ receptors in dendritic spines of CA1 hippocampal neurons plays a role in LTP induction. Notably, activation of these receptors reduces depolarization that is needed to remove Mg^2+^ block of NMDA receptors, thus leading to reduced LTP. Furthermore, a small population of GABAergic interneurons that express somatostatin makes synapses targeting directly on dendritic spine heads in the mouse mPFC (Chiu et al., 2013), being able to control local postsynaptic Ca^2+^ fluxes within the spines. All these different mechanisms indicate that the GABA_A_ receptor activity may regulate rapid dynamics and long-term structural events in glutamate synapses.

The strength of GABAergic synapses is determined by the size of releasable pool of presynaptic GABA and number and/or diversity of GABA_A_ receptors sitting on postsynaptic membrane, which, in turn, is largely determined by receptor trafficking to and from the plasma membrane, including receptor insertion, lateral diffusion within membrane, removal, recycling and degradation (Vithlani et al., 2011). Interestingly, high-resolution two-photon imaging in organotypic hippocampal cultures has revealed that new GABAergic synapses are formed by the appearance of new boutons at pre-existing axon-dendrite crossings (Wierenga et al., 2008). Hence, in that model, plasticity in GABAergic synapses depends on the number of available axon-dendrite crossings, which makes it more restricted than plasticity sites in glutamatergic connections. However, it is not known yet whether these *in vitro* results can be generalized to all GABAergic synapses, and, therefore, further *in vivo* studies are needed.

## DRUG-INDUCED PLASTICITY OF GABAergic SYNAPSES

In addition to fast modulation of channel gating, both BZs and neurosteroids have longer lasting effects by controlling the number and subtypes of GABA_A_ receptors on the plasma membrane (Vithlani et al., 2011; Deeb et al., 2012; Abramian et al., 2014). Notably, treatment of hippocampal cultures with BZ agonist flurazepam modulates GABA_A_ receptor trafficking by promoting selective degradation of α2 subunit-containing GABA_A_ receptors after their removal from the postsynaptic membrane, leading to a reduction in synapse size and number, and finally to depression of synaptic inhibition (Jacob et al., 2012). The development of tolerance to the sedative effects of diazepam is associated with a decrease in [^3^H]L-655,708 binding to the hippocampal dentate gyrus α5 subunit-containing receptors (dependent on α1 subunit-containing receptors), an effect which was extended by the absence of tolerance in α5 subunit point-mutant mice (van Rijnsoever et al., 2004). Treatment of *C. elegans* with the GABA-site agonist muscimol also results in selective removal of GABA_A_ receptors from synapses (Davis et al., 2010). In contrast, neurosteroids can selectively enhance the trafficking of extrasynaptic GABA_A_ receptors by insertion of new α4 subunit-containing GABA_A_ receptors into the membrane, resulting in an enhancement of tonic inhibition in mice (Abramian et al., 2014). Thus, the neuronal adaptations to GABA_A_ drugs via modulation of receptor trafficking produce long-lasting changes in the efficacy of GABAergic inhibition. However, it should be noted that the effects of GABA_A_ ligands on receptor subunits are very much dependent on the experimental model, and the effects seen in cell culture models have often been difficult to reproduce *in vivo* (Uusi-Oukari and Korpi, 2010).

The most obvious impediment to understanding neuroadaptation induced by GABA_A_ receptor modulators is the diversity of their target neurons. In fact, each neuron controlled by inhibitory terminals expresses its unique combination of GABA_A_ receptors (Luddens et al., 1995; Olsen and Sieghart, 2009). A further obstacle to the studying of neuronal and structural drug-induced plasticity is made by the fact that interneurons, as a major target for GABA_A_ receptor modulators, themselves are innervated by both glutamatergic and GABAergic synapses. In addition, endogenous GABA_A_ receptor modulators, such as neurosteroids, whose levels constantly fluctuate, e.g., during stress, menstrual cycle and development, can directly affect memory and learning processing (Maguire et al., 2005; Shen et al., 2010), as well as influence the action of other GABA_A_ receptor modulators.

## KCC2-MEDIATED SPINE MORPHOGENESIS

Potassium-chloride co-transporter 2 (KCC2) is expressed in neurons to create the driving force for chloride ions to travel into the cell through the GABA_A_ receptor anion channel, which then leads to hyperpolarizing GABA actions (Rivera et al., 1999). Independently of its Cl^-^ transport function KCC2 has also gained attention due to its structural role in both glutamatergic and GABAergic synapses (Li et al., 2007; Horn et al., 2010; Sun et al., 2013). In glutamatergic synapses, KCC2 located in the neck and head of dendritic spines binds to actin cytoskeleton via the linker protein 4.1 N (Li et al., 2007). While the exact molecular mechanisms still remain elusive, the interaction of KCC2 with the cytoskeleton is crucial for the maturation of spines and for the stability of AMPA receptor clusters (Li et al., 2007; Gauvain et al., 2011). In contrast to glutamatergic synapses, GABAergic synapses are usually located directly on the dendritic shaft (Freund and Buzsaki, 1996; Somogyi et al., 1998). KCC2 expression is regulated by a cell adhesion molecule neuroligin-2 which is mostly localized at GABAergic synapses. Knockdown of neuroligin-2 down-regulates expression of KCC2 and reduces GABAergic synaptogenesis, and interestingly, by affecting the KCC2 levels it also down-regulates the number of glutamatergic synapses (Sun et al., 2013).

Although there is no direct evidence yet on drug-induced structural changes through KCC2-dependent mechanisms, several studies demonstrated changes in expression levels of KCC2. Chronic treatment with the BZ agonist zolpidem up-regulated the KCC2 expression in mouse limbic forebrain (Shibasaki et al., 2013). Also the neurosteroid allopregnanolone transiently modifies KCC2 expression and protein levels during brain maturation in male rats (Modol et al., 2014). However, treatment with general anesthetics, midazolam, propofol, and ketamine, does not alter the expression of KCC2 in rats during the first two postnatal weeks when developmental maturation of KCC2 expression is going on (Lacoh et al., 2013). Future studies should be directed to clarify whether KCC2-mediated mechanisms play a role in neuronal plasticity induced by GABAergic drugs (Kang et al., 2006), and which particular isoforms of KCC2 play role in structural changes, since a recent study suggests that KCC2a and KCC2b isoforms have different brain regional distributions and likely different roles in neuronal functions (Markkanen et al., 2014).

## DRUG-INDUCED SYNAPTIC PLASTICITY IN THE VTA

The VTA has been widely studied given its fundamental role in motivation and reward (for review, see Luscher and Malenka, 2011). VTA DA neurons project mainly to the NAc and mPFC and less extensively to the hippocampus and amygdala. They receive glutamatergic inputs from many brain regions, including the mPFC, lateral hypothalamus, lateral habenula and hippocampus, and in addition to local inhibitory control from the VTA GABAergic interneurons, GABAergic inputs to DA neurons arise from the NAc, ventral pallidum, nuclei of the extended amygdala, and rostromedial tegmental nucleus (Jhou et al., 2009; Omelchenko and Sesack, 2009; Watabe-Uchida et al., 2012).

Glutamatergic transmission in the VTA is critical to the reinforcing effects of drugs of abuse: suppressing the glutamatergic transmission in the VTA attenuates cocaine and heroin reward (Xi and Stein, 2002; You et al., 2007) and prevents the reinstatement of cocaine- or heroin seeking (Bossert et al., 2004; Sun et al., 2005). Glutamatergic synapses on VTA DA neurons can undergo both NMDAR-dependent LTP and NMDAR-independent long-term depression (LTD; Jones et al., 2000; Thomas and Malenka, 2003). Synaptic plasticity in the mesolimbic DA system was early on hypothesized to play a role in the process of drug reinforcement and addiction. During the last decade, evidence from electrophysiological studies have accumulated showing that in addition to acute activation of VTA DA neurons, drugs of abuse also induce long-lasting plasticity in the synapses of these neurons. Several classical drugs of abuse such as cocaine, amphetamine, morphine, nicotine, and ethanol share the ability to induce an NMDAR-dependent LTP at glutamatergic synapses of VTA DA neurons via insertion of new GluA2 subunit-lacking AMPARs (Ungless et al., 2001; Saal et al., 2003; Luscher and Malenka, 2011).

In addition to the potentiation of glutamatergic transmission, different addictive drugs such as morphine, nicotine, cocaine, and ethanol have been found to impair GABAergic transmission in the VTA DA neurons by preventing the LTP of GABAergic synapses (LTP_GABA_; Nugent et al., 2007; Guan and Ye, 2010). In the VTA, LTP_GABA_ is triggered by NMDA receptor activation at glutamate synapses and requires nitric oxide-cGMP signaling (Nugent et al., 2007; Nugent and Kauer, 2008). Blockade of LTP_GABA_ could additionally increase release of DA by silencing local GABA neurons (Liu et al., 2000; Nugent and Kauer, 2008; Niehaus et al., 2010). Which particular GABAergic inputs are involved in LTP_GABA_ and whether the GABA_A_ receptor modulators induce LTP_GABA_ have not been investigated so far.

## GABA_A_ RECEPTOR BENZODIAZEPINE-SITE DRUGS INDUCE NEURONAL PLASTICITY IN THE VTA

Since the positive modulators of GABA_A_ receptor benzodiazepine site have well-known abuse potential and act as positive and/or negative (Panlilio et al., 2005) reinforcers in different animal models of addiction, their effects on synaptic plasticity in VTA DA neurons have been recently studied. Indeed, diazepam, and zolpidem, similarly to the other drugs of abuse, were shown to induce plasticity in the glutamatergic synapses contacting VTA DA neurons (Heikkinen et al., 2009). Particularly, BZs induced an LTP that was prevented by co-administration of the BZ antagonist flumazenil and by the NMDA receptor antagonist dizocilpine (MK-801; Heikkinen et al., 2009). BZ-induced LTP in VTA DA neurons was associated with insertion of new GluA2-lacking AMPA receptors, and intra-VTA local network was sufficient for the LTP induction via inhibition of VTA GABAergic interneurons (Tan et al., 2010). Furthermore, Tan et al. (2010) examined a mutant mouse with BZ-insensitive α1 subunits, and found that in these mice midazolam was not able to inhibit the firing of VTA GABAergic interneurons, to disinhibit the DA neurons, to induce plasticity at glutamatergic synapses or to support drug-reinforcement behavior. This suggests that the BZ-induced disinhibition of DA neurons is a key mechanism involved in BZ-induced plasticity in VTA DA neurons as well as in BZ reinforcement.

## DRUGS TARGETING THE EXTRASYNAPTIC GABA_A_ RECEPTORS INDUCE VTA DA NEURON PLASTICITY BUT ARE AVERSIVE

There is a need for new anxiolytic/sedative drugs with no abuse potential. One approach has been to target a GABAergic system separate from the benzodiazepine-sensitive GABA_A_ receptors, i.e., the extrasynaptic GABA_A_ receptors containing δ-subunit (Olsen and Sieghart, 2009). However, δ-subunit is expressed along the reward pathway in the VTA, NAc, mPFC, and hippocampus (Pirker et al., 2000; Hortnagl et al., 2013). THIP and muscimol, which act on GABA_A_ receptor agonist sites with preferential activation of the high-affinity extrasynaptic receptors (Chandra et al., 2010), have been shown to increase firing rates of DA neurons (Waszczak and Walters, 1980). Thus, it was necessary to study the effects of modulators of the extrasynaptic GABA_A_ system on plasticity in reward pathway and the possible reinforcing potential of these drugs.

A single dose of THIP and another extrasynaptic GABA_A_ receptor modulator neurosteroid ganaxolone dose-dependently induced similar AMPA receptor-mediated LTP in VTA DA neurons via the primary action of increased tonic inhibition of VTA GABAergic interneurons (Vashchinkina et al., 2012, 2014). Importantly, both THIP- and ganaxolone-induced plasticity lasted at least for six days, while BZs cause this effect for three days only (Heikkinen et al., 2009). The effects of THIP and ganaxolone were absent in δ-GABA_A_ receptor knockout mice, and both treatments enhanced AMPA current rectification, indicating reduced targeting of GluA2 subunits.

Surprisingly, despite of aforementioned similar effects on neuroadaptations in VTA DA neurons, modulators of synaptic, and extrasynaptic GABA_A_ receptors induce distinct behavior in the drug self-administration and place conditioning paradigms which have been previously associated with activity of VTA DA neurons (for review, see Luscher and Malenka, 2011). While activation of the synaptic α1 subunit-containing GABA_A_ receptors in the VTA by oral midazolam is reinforcing in the self-administration, the activation of extrasynaptic receptors by ip THIP or ganaxolone leads to avoidance behavior as seen in conditioned place aversion, and THIP is not self-administered either by mice or baboons (Tan et al., 2010; Vashchinkina et al., 2012, 2014). These aversive effects were abolished in GABA_A_ receptor δ subunit-deficient mice (Vashchinkina et al., 2014), suggesting a specific role of this receptor population in the VTA. These behavioral findings support the hypothesis that activation of synaptic and extrasynaptic GABA_A_ receptors is rewarding and aversive, respectively. This may depend on the primary brain areas targeted, as illustrated by intracerebral injections of muscimol (see below) that also preferentially targets extrasynaptic receptors (Chandra et al., 2010). By using place-conditioning system, muscimol infusion into the NAc shell provoked conditioned place preference when infused anteriorly and conditioned aversion when infused posteriorly (Reynolds and Berridge, 2002). Intra-BLA infusions of muscimol and bicuculline had no effect on reward (Zarrindast et al., 2004; Macedo et al., 2006).

Furthermore, it is now becoming clear that aversive drugs or experiences can acutely activate certain DA neurons in the VTA and also induce a long-lasting potentiation at their glutamatergic synapses. This was actually reported already in the early paper of Saal et al. (2003) where the authors showed that in mice a 5-min swimming stress at 6°C water bath induces similar plasticity in the VTA DA neurons as the classical drugs of abuse.

## DO GABA_A_ RECEPTOR DRUGS TARGET THE SAME POPULATIONS OF VTA DA NEURONS?

DA neurons are divergent in many respects, e.g., in their electrophysiological features, vulnerability to neurodegeneration and regulation by neuropeptides (Korotkova et al., 2004; Lammel et al., 2011). In particular, Lammel et al. (2011) have shown that DA neurons in the mouse VTA are organized into anatomical and electrophysiological subpopulations inside the DAergic nuclei depending on their projection terminal fields. However, in rats, the VTA neurons might be more heterogeneously organized (Margolis et al., 2006, 2012). The inhibitory control from VTA GABA interneurons is an important regulator of VTA DA neuron activity and the following behavioral outcome. Aversive stimuli have been shown to increase the firing of VTA GABA neurons (Creed et al., 2014). However, while GABA neurons in the VTA seem to quite faithfully respond to aversion by excitation, the responses in VTA DA neurons are more heterogeneous: activation, no response or inhibition have been observed in monkeys (Matsumoto and Hikosaka, 2009). Rewarding or aversive stimuli might modulate the activity of DA neurons differently depending on the brain area to which these neurons project. In mice, a cocaine experience selectively affected DA cells projecting to the NAc medial shell, while an aversive stimulus influenced DA cells projecting to the PFC, and the DA neurons projecting to the NAc lateral shell were modified by both rewarding and aversive stimuli, suggesting that the mesocorticolimbic DA system is comprised of anatomically distinct circuits, modified by different motivational relevance (Lammel et al., 2011). In anesthetized rats, foot shock inhibited DA neurons in the dorsal VTA, whereas the DA neurons in the ventral VTA became phasically excited (Brischoux et al., 2009). In mice, the majority of the VTA DA neurons decreased firing under fearful events, but a small group of DA neurons were activated (Wang and Tsien, 2011). Another study reported that a similar number of DA neurons were activated, inhibited or unaltered by tail pinch, and it also showed that in mice with an impaired NMDA receptor-mediated control of DA neurons the DAergic activation in response to an aversive stimulus was attenuated, leading to impaired aversive conditioning (Zweifel et al., 2011). These findings suggest that increases in DA signaling can be evoked by stimuli with motivational relevance to either rewarding or aversive direction, and they point toward putative multiple populations of VTA DA neurons with different afferent and efferent connections.

Mouse DA neurons with pronounced hyperpolarization-activated cation current *I*_h_-current are found in the lateral VTA and they project to the lateral NAc shell, while the DA neurons of the medial posterior VTA project to the mPFC and medial NAc shell, and have no or very small *I*_h_-currents (Lammel et al., 2011). It should be noted that the study revealing BZ-induced glutamatergic plasticity in VTA DA neurons used a large *I*_h_-current as a marker for DA neurons (Heikkinen et al., 2009). Thus, mostly a subpopulation of VTA DA neurons that projected to the lateral NAc shell was studied. In the studies with THIP and ganaxolone, a genetically modified mouse line with a fluorescent protein marker expressed in tyrosine hydroxylase-positive neurons was used (Vashchinkina et al., 2012, 2014). This allowed recording also from the DA neurons in more medial areas of the VTA with small or no *I*_h_-currents and projecting to the mPFC. It is possible that BZs are positively reinforcing due to mainly activating and modifying the DA neurons detecting reward whereas the drugs activating the extrasynaptic GABA_A_ receptors lead to conditioned aversion because they activate and induce LTP in the DA neurons involved in negative motivation. This hypothesis remains to be carefully tested, although the post-study examination of the recording sites for DA neuron plasticity by THIP failed to indicate any anatomical localization within the VTA (Vashchinkina et al., 2012).

Both BZs and extrasynaptic GABA_A_ modulators produce strong inhibition of the VTA GABA interneurons (Heikkinen et al., 2009; Tan et al., 2010; Vashchinkina et al., 2012, 2014). Thus, a similar disinhibitory mechanism is believed to induce the activation and persistent modulation of VTA DA neurons by both classes of GABA_A_ drugs, which in turn suggests that they might be targeting different populations of VTA GABA interneurons enriched with synaptic and/or extrasynaptic GABA_A_ receptors. Interestingly, a recent study shows that the volatile solvent toluene, that also is a positive modulator of GABA_A_ receptors, induces LTP at the glutamatergic synapses of VTA DA neurons that project to NAc core and shell, whereas it failed to affect the synapses of mPFC-projecting neurons (Beckley et al., 2013). Importantly, toluene is abused by humans, and in rodent models of addiction it increases firing of VTA DA neurons and DA release in the NAc and exhibits positive reinforcement (Lee et al., 2006; Riegel et al., 2007; Lubman et al., 2008). Effects of toluene on GABA interneurons have not been studied so far.

In summary, the possible VTA heterogeneity of both the principal DA neurons and the GABA interneurons and projection neurons in responding to and mediating stimuli and affecting behaviors is an important subject for future research. This information would be needed to further develop rational treatment ideas for addiction.

## DRUG-INDUCED STRUCTURAL PLASTICITY IN THE VTA AND ITS EFFERENTS

Midbrain (VTA and substantia nigra) and striatal (NAc and the dorsomedial and dorsolateral striatae) components of the basal ganglia and mesolimbic pathway are connected in a spiraling manner so that ventral striatal regions project to medial parts of the midbrain DAergic area, which subsequently sends projections to more dorsal striatal regions which in turn project to the more lateral midbrain and so on (Haber et al., 2000). An appealing emerging hypothesis is that the initial drug-induced plasticity in the DAergic midbrain and subsequently in the ventral striatum would recruit more and more dorsal striatal regions during chronic drug use and reinforce the connectivity within these spiral projections, thus leading to compulsive drug-seeking manifesting at the late stages of addiction (Grueter et al., 2012). The dorsomedial and the dorsolateral parts of striatum regulate goal-directed and stimulus–response habitual movements, respectively (Yin and Knowlton, 2006; Redgrave et al., 2010). The majority of the neurons of ventral and dorsal striatum are projecting medium spiny neurons (MSNs), whose activity depends to a great extent on excitatory inputs from cortical and limbic regions (Sesack and Grace, 2010). Plasticity at excitatory synapses of the striatum would thus change the output of striatal circuits, i.e., the motivated as well as compulsive behaviors. Addiction seems to involve exceptionally intense drug experience-driven synaptic and structural plasticity at different levels of the mesolimbic DA system (Robinson and Kolb, 1999).

**FIGURE 1:**
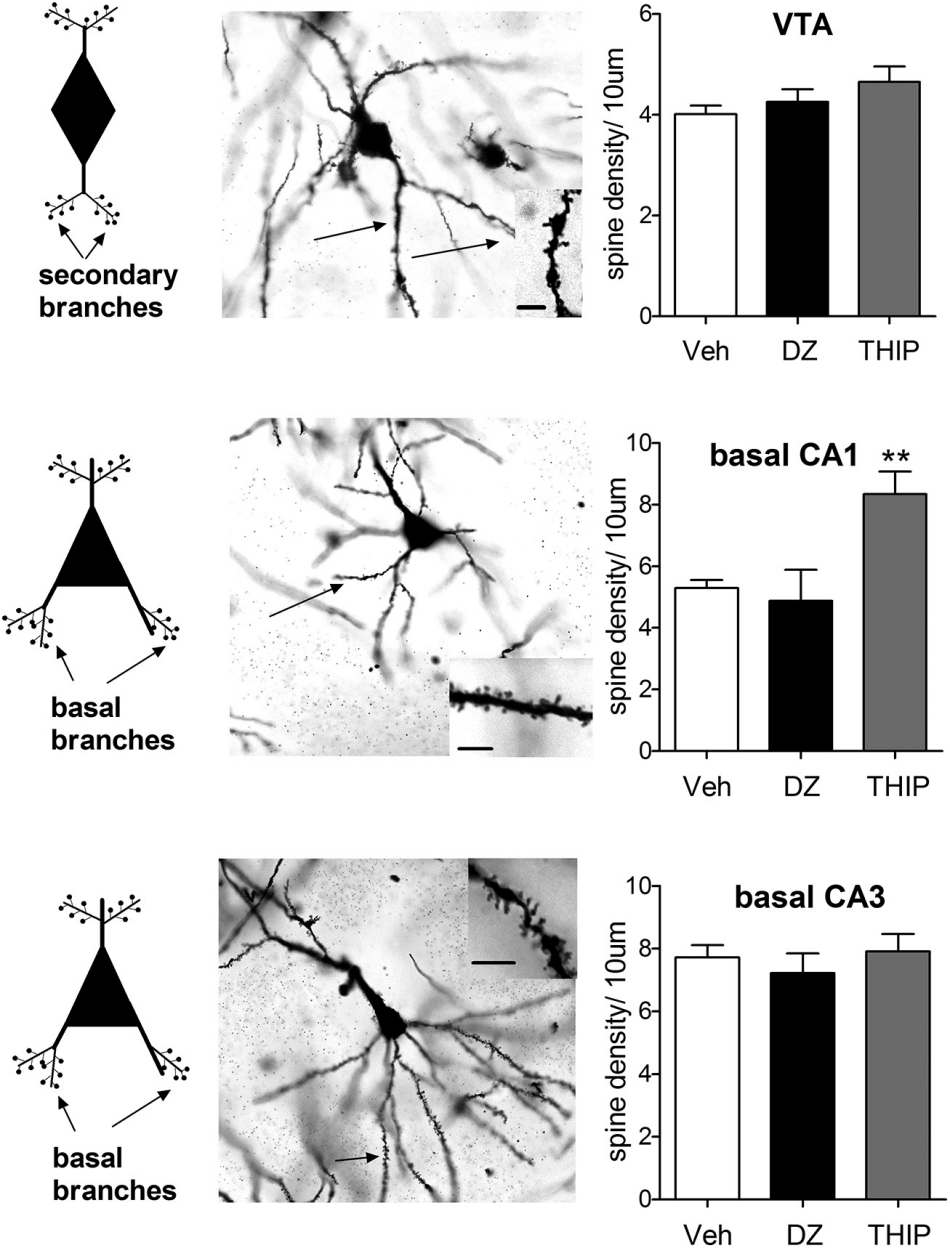
**Pyramidal cells of hippocampal CA1 region exhibit distinct morphological responses to diazepam and THIP treatment.** THIP increased the spine density on the basal secondary dendrites of CA1 pyramidal cells (*F*_2,15_ = 9.69, *p* < 0.01), but not in CA3 or VTA (*p* > 0.05). In contrast, diazepam (DZ) did not show any changes in spine density of the basal nor apical dendritic spines in these areas (*p* > 0.05). *On the left,* schematic neurons depict the spines either secondary or basal dendritic branches that were assessed here. *In the middle*, representative images of Golgi-stained neurons on horizontal sections. Insets are dendrites showing spines. Scale bar is 10 μm. *On the right*, plots showing the spine densities 24 h after a single injection of DZ or THIP. Mean ± SEM for *n* = 10, 5, and 5 mice vehicle (Veh), DZ, and THIP groups, respectively. **p < 0.01, (one-way ANOVA followed by Bonferroni *post hoc* test). C57BL/6J mice (21–26 days old) were decapitated at 24 h after treatment with DZ (5 mg/kg, ip), THIP (6 mg/kg, ip) or vehicle (Heikkinen et al., 2009; Vashchinkina et al., 2012; All animal procedures were approved by the Southern Finland Provincial Government). Brains were rapidly dissected and immediately placed into Golgi-Cox fixative and processed using the FD Rapid GolgiStain Kit (FD Neuro Technologies). Impregnated brains were then cut in serial horizontal 80-μm-thick sections using a cryostat (Leica Microsystems, Nussloch, Germany) and stained using the kit protocol. For each brain area, spine density per 10 μm was assessed in five cells. Notably, in VTA cells spines were determined on secondary dendrites, in CA1 and CA3 pyramidal cells on basal dendritic branches, using the 100× objective of Zeiss Axioplan microscope (Carl Zeiss MicroImaging GmbH, NA = 1.3, Ph3, DIC, oil), and later analyzed by National Institutes of Health ImageJ software (http://rsb.info.nih.gov/ij). Spine counting was performed blind concerning the treatments. Only protrusions that show continuity with the dendritic shaft were considered as spines.

GABA_A_ receptor modulators are abused, but to date there are no data available whether they are able to induce changes in neuronal spine density in the reward system. Earlier work by Sarti et al. (2007) demonstrated a correlation of cocaine-induced plasticity of glutamatergic synapses with an increase in spine density of the rat VTA neurons. In that work, the traditional Golgi-Cox impregnation was used to study dendritic spines and cell morphology was used to identify and subtype VTA DA neurons. Since GABA_A_ receptor modulators also induce similar glutamate receptor neuroplasticity in VTA DA neurons, we were puzzled with a question whether diazepam and/or THIP alter morphology and spine density. At 24 h after single injections of the doses of diazepam and THIP, which induce LTP in the VTA (Heikkinen et al., 2009; Vashchinkina et al., 2012), the brains of mice were subjected to Golgi staining and spine counting (**Figure 1**). In addition to the VTA, we analyzed the hippocampal CA1 and CA3 regions, since BZs are known to induce persistent synaptic plasticity particularly in the pyramidal neurons of the CA1 and CA3 regions (**Table 1**).

**Table 1:** Summary of neuroplasticity induced by GABA_A_ receptor drugs.

Brain area, cell type (species)	Drug treatment	Method	Main findings	Reference
**Ventral tegmental area**
Dopamine neurons (mouse)	24–120 h after DZ (5 mg/kg), THIP (6 mg/kg), ganaxolone (30 mg/kg), zolpidem (5 mg/kg), midazolam (0.5 mg/kg)	Electrophysiology	Insertion of new GluA2-lacking AMPA-Rs	Heikkinen et al. (2009), Tan et al. (2010), Vashchinkina et al. (2012, 2014)
**Nucleus accumbens**
			No data available	
**Cortex**
Layer V pyramidal neurons (rat)	96 h after diazepam for 14 days at increasing doses (17.6–70.4 μmol/kg)	IHC with gold-immunolabeling	Up-regulation of GluA1 subunit of AMPA-R’s mRNA and cognate protein	Izzo et al. (2001)
**Amygdala**
Neurons of lateral nucleus (mouse)	Short-term deep isoflurane anesthesia	Electrophysiology	Enhancement of capsaicin-induced LTP via TRPV1-mediated mechanism	Zschenderlein et al. (2011)
**Hippocampus**
CA1 pyramidal neurons (rat)	Withdrawal 96 h after DZ for 14 days at increasing doses (17.6–70.4 μmol/kg)	IHC with gold-immunolabeling	Up-regulation of GluA1 subunit of AMPA-R’s mRNA and cognate protein	Izzo et al. (2001)
CA1 pyramidal neurons (rat)	Flurazepam-induced DZ withdrawal	Electrophysiology	Up-regulation of AMPA-R function	Van Sickle et al. (2004), Song et al. (2007), Xiang and Tietz (2007)
CA1, CA3 pyramidal neurons, granule layer of DG (rat)	Acute *in vitro* (5 μM) and *in vivo* DZ and triazolam	Electrophysiology	Inhibition of LTP	del Cerro et al. (1992), Mori et al. (2001), Maubach et al. (2004), Xu and Sastry (2005)
CA1 pyramidal neurons (mouse)	*In vitro* isoflurane (0.125-0.5 mM)	Electrophysiology	Inhibition of LTD and LTP in GABA_A_R- dependent manner, nAChR α4β2 subtype-mediated mechanism	Simon et al. (2001), Haseneder et al. (2009)
CA1 pyramidal neurons (mouse)	24 h–7 day after 2 h – isoflurane anesthesia	Electrophysiology, Western blotting	Enhancement of LTP via up-regulation of GluN2B subunit of NMDA-Rs	Rammes et al. (2009)

*AMPA-R, α-amino-3-hydroxy-5-methyl-4-isoxazolepropionic acid receptor; CA, *Cornu Ammonis,* hippocampal areas; DG, dentate gyrus; DZ, diazepam; IHC, immunohistochemistry; nAChR, nicotinic acetylcholine receptor; NMDA-R, *N*-methyl-D-aspartate receptor; THIP, gaboxadol.*

We found that basal spine density counts in both hippocampal and VTA neurons are in line with the results of (Sarti et al., 2007). Treatment with THIP increased the spine density on the basal secondary dendrites of CA1 pyramidal cells (**Figure 1**), but not in CA3 or VTA. In contrast, diazepam treatment did not show any changes in spine density of the basal dendritic spines in these areas, nor in the apical ones (data not shown). This is generally consistent with a previous study, in which acute and chronic treatment with BZs was not associated with up-regulation of BDNF or c-Fos protein levels in the hippocampus (Licata et al., 2013). Since Sarti et al. (2007) found clear increase in VTA spines after neuroplasticity-inducing dose of cocaine, our results suggest different mechanisms or different VTA DA neuron populations might have been involved in the effects of various GABA_A_ ligands. Additional studies using retrograde neurotracers to clarify the targets and novel immunofluorescent co-staining methods for precise phenotyping of the neurons (Spiga et al., 2011) are needed to resolve the affected VTA neurons. In spite of the comparable VTA neuroplasticity-inducing doses used, diazepam and THIP produced distinct effects on spine remodeling. These results show that the acute effects of GABA-drugs are not consistently accompanied by changes in spine densities of the dendrites in hippocampal or VTA neurons.

Repeated exposure to cocaine has been shown to increase the number of silent synapses and the density of dendritic spines in NAc shell (Robinson and Kolb, 1999; Huang et al., 2009; Dobi et al., 2011; Kim et al., 2011). Also dorsal striatum MSNs exhibit an increased dendritic spine density following chronic cocaine exposure (Ren et al., 2010). Months after repeated exposure to methamphetamine the spine density increased in MSNs of the dorsolateral striatum, a structure that supports habitual behaviors but decreased in dorsomedial striatum which is important in goal-directed movements (Jedynak et al., 2007). A primate study of chronic ethanol drinking reported increased spine density in the putamen (the primate analog of dorsolateral striatum in rodents) as well as enhanced glutamatergic transmission and increased intrinsic excitability of MSNs in this area (Cuzon Carlson et al., 2011). This pattern of structural plasticity in the dorsal striatum supports the concept that during the progression of addiction the behavior is driven toward habitual drug taking and seeking.

Withdrawal from chronic treatment with addictive drugs leads to hypofunction of VTA DA neurons. For example, withdrawal from cannabinoids or morphine profoundly affects the morphological characteristics of VTA DA neurons and spine density of MSNs of the NAc shell (Spiga et al., 2003, 2010). Whether chronic treatment with GABA_A_ receptor modulators alters morphology of VTA DA neurons or NAc and dorsal striatal MSNs, with respect to addiction and/or withdrawal, remains to be studied.

Broad distribution of GABA_A_ receptors throughout the brain suggests more widespread neuroplasticity effects of GABA_A_ receptor modulators in other brain regions such as the NAc, mPFC, hippocampus, and amygdala (Licata and Rowlett, 2008). In fact, several reports showed blockade of hippocampal LTP after acute *in vitro* effects of BZs and isoflurane (summarized in **Table 1**). This is consistent with the known effect of cognitive dysfunction induced by general anesthetics, and memory impairment by BZs in humans (Canet et al., 2003; Newman et al., 2007). On the other hand, withdrawal symptoms after repeated administration of these drugs are associated with synthesis of new glutamatergic receptors and potentiation of LTP in the hippocampus and cortex (**Table 1**). Thus, the glutamatergic receptors appear to be regulated differently depending on the specific phase of the drug effect.

## CONCLUSION

GABAergic neurotransmission is known to participate in neuronal plasticity processes during the critical periods in development, and drugs acting on GABA_A_ receptors, such as BZs, have been considered more as blunting neuroplasticity than inducing it. However, recent experiments on the midbrain dopamine systems have revealed that GABA_A_ drugs acting on different receptor subtypes induce persistent neuroplasticity in glutamate receptors of the VTA DA neurons, but so far there is little evidence in support of widespread structural changes caused by these drugs. The main mechanism of how the GABA_A_ drugs induce plasticity involves disinhibition via primary inhibition of GABAergic interneurons. It is likely that more dynamic methods, such as *in vivo* microscopy on dendritic spines, will be needed to better understand the structural neuroplasticity/neurotoxicity effects of general anesthetics, BZs and other types of anxiolytics and sedatives. Based on the present data, the selection of an anesthetic agent will be important for these future experiments!

## AUTHOR CONTRIBUTIONS

Elena Vashchinkina, Anne Panhelainen, Teemu Aitta-aho, and Esa R. Korpi conceived and designed the experiments, Elena Vashchinkina performed the experiments and data analyses, and Elena Vashchinkina, Anne Panhelainen, Teemu Aitta-aho, and Esa R. Korpi wrote the paper.

## Conflict of Interest Statement

The authors declare that the research was conducted in the absence of any commercial or financial relationships that could be construed as a potential conflict of interest.
